# Analysis of multivariate longitudinal substance use outcomes using multivariate mixed cumulative logit model

**DOI:** 10.1186/s12874-021-01444-1

**Published:** 2021-11-06

**Authors:** Xiaolei Lin, Robin Mermelstein, Donald Hedeker

**Affiliations:** 1 School of Data Science, Fudan University, Shanghai, China; 2 Institute for Health Research and Policy, University of Illinois at Chicago, Chicago, USA; 3 Department of Public Health Sciences, University of Chicago, Chicago, USA

**Keywords:** Mixed cumulative logit model, Multivariate longitudinal outcomes, Non-proportional odds assumption, Substance usage

## Abstract

**Abstract:**

**Background:**

Longitudinal assessments of usage are often conducted for multiple substances (e.g., cigarettes, alcohol and marijuana) and research interests are often focused on the inter-substance association. We propose a multivariate longitudinal modeling approach for jointly analyzing the ordinal multivariate substance use data.

**Methods:**

We describe how the binary random slope logistic regression model can be extended to the multi-category ordinal outcomes. We also describe how the proportional odds assumption can be relaxed by allowing differential covariate effects on different cumulative logits for multiple outcomes. Data are analyzed from a P01 study that evaluates the usage levels of cigarettes, alcohol and marijuana repeatedly across 8 measurement waves during 7 consecutive years.

**Results:**

1263 subjects participated in the study with informed consent, among whom 56.6% are females. Males and females show significant differences in terms of the time trend for substance use. Specifically, males showed steeper trends on cigarette and marijuana use over time compared to females, while less so for alcohol. For all three substances, age effects appear to be different for different cumulative logits, indicating the violation of proportional odds assumption.

**Conclusions:**

The multivariate mixed cumulative logit model offers the most flexibility and allows one to examine the inter-substance association when proportional odds assumption is violated.

**Supplementary Information:**

The online version contains supplementary material available at 10.1186/s12874-021-01444-1.

## Background

Usage levels of multiple substances (e.g., cigarettes, alcohol and marijuana) are often collected together and repeatedly over time [1]. These longitudinal outcomes may be modeled using univariate approaches, such as univariate mixed effect models or univariate generalized estimating equations [2, 3]. However, research questions often arise in investigating the inter-substance association of these multiple substances and therefore a multivariate longitudinal model offers a more desirable alternative. The multivariate longitudinal approach allows the test of whether increases / decreases in use of one substance are associated with increases / decreases in another substance of interest, or the test of whether a potential intervention effect is the same / different across multiple substances [4].

A major challenge for the multivariate longitudinal approach is that the measurement scales of these products may be different. For example, the usage level of cigarette may be collected in terms of the number of cigarettes smoked per day, while frequency of binge drinking per week for alcohol. A practical way to “standardize” these measurement scales is to treat them as ordinal [5–7]. Specifically, the usage levels of these products can be summarized in terms of no, low, moderate, and high use, corresponding to, for example, 0, 1–3, 4–20, 20+ days of use within the last 30 days. Therefore, a multivariate modeling approach for ordinal longitudinal data will be considered.

Ordinal models characterize the cumulative comparisons of usage levels, i.e., no vs any use, no and low use vs moderate and high use, no to moderate use vs high use [8]. It is common to assume that covariates have the same effect on these cumulative comparisons, which is often known as the proportional odds assumption. However, for substance use outcomes, this assumption may not be reasonable [9, 10]. For example, suppose that there are four categories as mentioned above (no, low, moderate and high use), a potential intervention may be successful in increasing the probability of moving from high to moderate use, but not from low to no use. That is to say, the effects of the intervention (i.e., the covariate) vary for different cumulative comparisons, where it would be observed when we compare no, low and moderate use vs high use, but would not be observed when we compare no vs any use. This flexibility allows covariate effects to vary across the lowest and highest levels of substance use and is unique to the non-proportional odds ordinal model. Thus, we believe that a longitudinal (non-proportional odds) ordinal model for multivariate outcomes is a viable approach for jointly modeling the usage levels for multiple substances.

Marginal models that focus on estimating the population averaged covariate effects, such as the Generalized Estimating Equations (GEE) can be used for longitudinal ordinal data. Heagerty and Zeger [11] extended the traditional GEE model (for continuous response) to accommodate correlated ordinal responses. Alternative to the class of marginal models, conditional models directly model the subject specific covariate effect using random effects. Hedeker and Gibbons [12] proposed a mixed effects model for analyzing ordinal longitudinal responses via probit and logistic link functions. Hedeker and Gibbons [13] then extended the model to accommodate multiple random effects to allow for both inter and intra-individual variations. Liu and Hedeker [14] extended the mixed effects item response theory model to allow for three-level multivariate ordinal outcomes without proportional odds assumption, but this model can only handle random intercept or item factor loading. In this paper, we describe and illustrate the use of an extended ordinal mixed model for analyzing multi-wave usage levels of multiple substances (cigarettes, alcohol and marijuana).

## Methods

For ordinal categorical outcomes, the mixed cumulative logit model is often constructed by first extending the binary logistic regression model to accommodate more than two categories, and then augmenting the cumulative logit model with subject level random effects. Parameter estimation in mixed cumulative logit models is computationally intensive since marginal likelihood needs to be integrated with respect to random effects and estimators are updated iteratively. In this article, we will focus on the application of the model rather than parameter estimation methods.

Suppose K (K ≥ 2) outcomes are repeatedly measured over time in a longitudinal study and each outcome has C ordinal levels. Let ${Y}_{ij}^k$ denote the k-th (k = 1, …, K) outcome for subject i (i = 1, …, N) on occasion j (j = 1, …, *n*_*i*_). In the simple case of multivariate binary outcomes, i.e., ${Y}_{ij}^k$ takes on values of either 0 or 1, mixed logistic regression model can be written in terms of the log odds of Pr(${Y}_{ij}^k=1$):

1 $${\log}\left[\frac{{\Pr}\left({Y}_{ij}^k=1\right)}{1-{\Pr}\left({Y}_{ij}^k=1\right)}\right]={\upbeta}_0^k+{\upbeta}_1^k\ {t}_{ij}+{\upbeta}_2^k\ {x}_i+{\upbeta}_3^k\ {x}_{ij}+{\upnu}_i^k+{\upmu}_i^k\ {t}_{ij}$$

In the left-hand side, the ratio $\frac{{\Pr}\left({Y}_{ij}^k=1\right)}{1-{\Pr}\left({Y}_{ij}^k=1\right)}$ is the odds of a “1” outcome, and its log ${\log}\left[\frac{{\Pr}\left({Y}_{ij}^k=1\right)}{1-{\Pr}\left({Y}_{ij}^k=1\right)}\right]$ is thus the log odds of a “1” outcome. This transformation of the probability ${\Pr}\left({Y}_{ij}^k=1\right)$ is also called the logit transformation. The log odds ${\log}\left[\frac{{\Pr}\left({Y}_{ij}^k=1\right)}{1-{\Pr}\left({Y}_{ij}^k=1\right)}\right]$ measures the possibility of a “1” outcome vs a “0” outcome, and is positive when ${\Pr}\left({Y}_{ij}^k=1\right)>0.5$, i.e., when “1” is more likely than “0”, negative vice versa. In terms of the regression coefficients, all β ‘s are superscripted with k, meaning that these are the coefficients for the k-th outcome, among which ${\upbeta}_0^k$ is the intercept, ${\upbeta}_1^k$ is the coefficient for time *t*_*ij*_, ${\upbeta}_2^k$ is the coefficient for the subject level covariate *x*_*i*_ (also called time-invariant covariate, e.g., gender), and ${\upbeta}_3^k$ is the coefficient for the occasion level covariate *x*_*ij*_ (also called time varying covariate, e.g., positive affect that can change during the study). The subject and occasion level covariates can be distinguished by their subscripts, i.e., whether the values vary by subject (subscripted by i) or across subject and occasion (subscripted by both i and j). Without loss of generality, the above model can incorporate more covariates - either at subject or occasion level, or interactions between any two covariates. The random effect vector $\left({\upnu}_i^k,{\upmu}_i^k\right)$ represents the effect of subject i on the log odds of a “1″ outcome at baseline occasion (*t*_*ij*_ = 0) and its change over time (slope), and is often assumed to follow a bivariate normal distribution with 0 mean vector and covariance Σ_νμ_ in univariate approach. For multivariate models, however, the multivariate random effects vector ${W}_i=\left({\upnu}_i^1,{\upmu}_i^1,{\upnu}_i^2,{\upmu}_i^2,\dots, {\upnu}_i^K,{\upmu}_i^K\right)$ is assumed to follow a 2 K dimensional multivariate normal distribution with 0 mean vector and a covariance matrix Σ_*w*_, allowing correlated random effects across different outcomes. It is assumed that $\left({\upnu}_i^k,{\upmu}_i^k\right)$ is representative of subject level characteristics that can be obtained from the repeated measurements. The randomness and distributional assumption of $\left({\upnu}_i^k,{\upmu}_i^k\right)$ or *W*_*i*_ separates the mixed effects models from fixed effects models, which treat $\left({\upnu}_i^k,{\upmu}_i^k\right)$ as model parameters (i.e., fixed instead of random) that can only be estimated using individual data. Model (1) is also called random slope logistic regression model because there are both random intercept and random slope in the model.

Extending Model (1) for ordinal outcome ${Y}_{ij}^k$ with a total of C + 1 (C ≥ 1) categories, we model the cumulative odds $\frac{{\Pr}\left({Y}_{ij}^k\le c\right)}{1-{\Pr}\left({Y}_{ij}^k\le c\right)}$ (c = 0, …, C-1) using multivariate mixed cumulative logit model:

2 $${\log}\left[\frac{{\Pr}\left({Y}_{ij}^k\le c\right)}{1-{\Pr}\left({Y}_{ij}^k\le c\right)}\right]={\upbeta}_{0c}^k+{\upbeta}_1^k\ {t}_{ij}+{\upbeta}_2^k\ {x}_i+{\upbeta}_3^k\ {x}_{ij}+{\upnu}_i^k+{\upmu}_i^k\ {t}_{ij}$$

for c = 0, …, C-1. The intercept ${\upbeta}_{0c}^k$ is now subscripted with c and is used to model the marginal frequencies in the C ordered categories. In Model (2), the cumulative odds of ${\Pr}\left({Y}_{ij}^k\le c\right)$ (rather than ${\Pr}\left({Y}_{ij}^k\ge c\right)$) is used, and thus positive values of the regression coefficients (${\upbeta}_1^k,{\upbeta}_2^k,{\upbeta}_3^k$) indicate a negative association between the outcome *Y*^*k*^ and corresponding covariate. That is to say, large values of *Y*^*k*^ is less likely to be observed with large values of the covariate. In the aforementioned example with 4 ordered categories, if no, low, moderate and heavy substance use is coded as 0, 1, 2 and 3 respectively, a positive coefficient would indicate that larger values of the covariate are more likely to be observed with lower usage levels. The bivariate random effects vector $\left({\upnu}_i^k,{\upmu}_i^k\right)$ characterizes the subject level deviation at baseline occasion and change across the follow-up occasions, and is assumed constant across the C-1 cumulative odds models. Instead of assuming that $\left({\upnu}_i^k,{\upmu}_i^k\right)$ is independent across K outcomes as in the univariate approach, Model (2) assumes that ${W}_i=\left({\upnu}_i^1,{\upmu}_i^1,{\upnu}_i^2,{\upmu}_i^2,\dots, {\upnu}_i^K,{\upmu}_i^K\right)$ follows the 2 K dimensional multivariate Gaussian distribution as described in Model (1).

Model (2) adopts the proportional odds assumption since the coefficient vector (${\upbeta}_1^k,{\upbeta}_2^k,{\upbeta}_3^k$) is constant across the C-1 cumulative comparisons, i.e., (${\upbeta}_1^k,{\upbeta}_2^k,{\upbeta}_3^k$) is not subscripted with c. This implies that effects of the covariates are the same across the C-1 cumulative comparisons. Suppose again that with 4 ordered categories, no, low, moderate and heavy substance use is coded as 0, 1, 2 and 3 respectively. We obtain the following cumulative logits model:

$${\log}\left[\frac{{\Pr}\left({Y}_{ij}^k\le 0\right)}{1-{\Pr}\left({Y}_{ij}^k\le 0\right)}\right]={\log}\left[\frac{{\Pr}\left({Y}_{ij}^k=0\right)}{{\Pr}\left({Y}_{ij}^k=1,2,3\right)}\right]={\upbeta}_{01}^k+{\upbeta}_1^k\ {t}_{ij}+{\upbeta}_2^k\ {x}_i+{\upbeta}_3^k\ {x}_{ij}+{\upnu}_i^k+{\upmu}_i^k\ {t}_{ij}$$

$${\log}\left[\frac{{\Pr}\left({Y}_{ij}^k\le 1\right)}{1-{\Pr}\left({Y}_{ij}^k\le 1\right)}\right]={\log}\left[\frac{{\Pr}\left({Y}_{ij}^k=0,1\right)}{{\Pr}\left({Y}_{ij}^k=2,3\right)}\right]={\upbeta}_{02}^k+{\upbeta}_1^k\ {t}_{ij}+{\upbeta}_2^k\ {x}_i+{\upbeta}_3^k\ {x}_{ij}+{\upnu}_i^k+{\upmu}_i^k\ {t}_{ij}$$

$${\log}\left[\frac{{\Pr}\left({Y}_{ij}^k\le 2\right)}{1-{\Pr}\left({Y}_{ij}^k\le 2\right)}\right]={\log}\left[\frac{{\Pr}\left({Y}_{ij}^k=0,1,2\right)}{{\Pr}\left({Y}_{ij}^k=3\right)}\right]={\upbeta}_{03}^k+{\upbeta}_1^k\ {t}_{ij}+{\upbeta}_2^k\ {x}_i+{\upbeta}_3^k\ {x}_{ij}+{\upnu}_i^k+{\upmu}_i^k\ {t}_{ij}$$

The above models imply that for one unit increase in time, the odds of being in a lower category (012 vs 3, 01 vs 23, and 0 vs 123) multiple by exp.(${\upbeta}_1^k$). As a result, there are 3 intercepts and only one set of regression coefficients to be estimated. Therefore, proportional odds assumption can greatly simplify the cumulative logit model by estimating a single effect for each covariate throughout all cumulative comparisons.

However, assuming homogeneous covariate effects for all cumulative logit models may not be reasonable in the context of substance use research. For example, a potential intervention might work by decreasing the odds of heavy use (thus increasing the odds of moderate use or levels below), while does not work well in increasing the odds of no use. In the above example, it is equivalent to say that β (covariate effect corresponding to the intervention) in the third equation (model for ${\log}\left[\frac{{\Pr}\left({Y}_{ij}^k=0,1,2\right)}{{\Pr}\left({Y}_{ij}^k=3\right)}\right]$) would be positive and significantly different from 0, while close to 0 in the first equation (model for ${\log}\left[\frac{{\Pr}\left({Y}_{ij}^k=0\right)}{{\Pr}\left({Y}_{ij}^k=1,2,3\right)}\right]$). In case of heterogeneous covariate effects across cumulative comparisons, mixed cumulative logit models that do not assume proportional odds will be considered. Peterson and Harrel [15] and Ierza [16] developed the ordinal models via logit and probit link functions with non-proportional odds for univariate cross-sectional data. Hedeker and Mermelstein [17] proposed the ordinal mixed logistic regression model with non-proportional odds for univariate longitudinal data. Extending the univariate models in Hedeker and Mermelstein, we propose a multivariate approach that is able to incorporate the correlation among multiple outcomes through random intercepts and slopes, as described in Model (3):

3 $${\log}\left[\frac{{\Pr}\left({Y}_{ij}^k\le c\right)}{1-{\Pr}\left({Y}_{ij}^k\le c\right)}\right]={\upbeta}_{0c}^k+{\upbeta}_{1c}^k\ {t}_{ij}+{\upbeta}_{2c}^k\ {x}_i+{\upbeta}_{3c}^k\ {x}_{ij}+{\upnu}_i^k+{\upmu}_i^k\ {t}_{ij}$$

for c = 0, …, C-1. The only difference of Model (3) compared to Model (2) is that the regression coefficients (${\upbeta}_{1c}^k,{\upbeta}_{2c}^k,{\upbeta}_{3c}^k$) now carry the c subscript and indicate the effect of the covariates on the c-th cumulative logits. In the above example of no, low, moderate and heavy use (coded as 0, 1, 2, 3) for the k-th substance, the non-proportional odds model becomes

$${\log}\left[\frac{{\Pr}\left({Y}_{ij}^k\le 0\right)}{1-{\Pr}\left({Y}_{ij}^k\le 0\right)}\right]={\log}\left[\frac{{\Pr}\left({Y}_{ij}^k=0\right)}{{\Pr}\left({Y}_{ij}^k=1,2,3\right)}\right]={\upbeta}_{00}^k+{\upbeta}_{10}^k\ {t}_{ij}+{\upbeta}_{20}^k\ {x}_i+{\upbeta}_{30}^k\ {x}_{ij}+{\upnu}_i^k+{\upmu}_i^k\ {t}_{ij}$$

$${\log}\left[\frac{{\Pr}\left({Y}_{ij}^k\le 1\right)}{1-{\Pr}\left({Y}_{ij}^k\le 1\right)}\right]={\log}\left[\frac{{\Pr}\left({Y}_{ij}^k=0,1\right)}{{\Pr}\left({Y}_{ij}^k=2,3\right)}\right]={\upbeta}_{01}^k+{\upbeta}_{11}^k\ {t}_{ij}+{\upbeta}_{21}^k\ {x}_i+{\upbeta}_{31}^k\ {x}_{ij}+{\upnu}_i^k+{\upmu}_i^k\ {t}_{ij}$$

$${\log}\left[\frac{{\Pr}\left({Y}_{ij}^k\le 2\right)}{1-{\Pr}\left({Y}_{ij}^k\le 2\right)}\right]={\log}\left[\frac{{\Pr}\left({Y}_{ij}^k=0,1,2\right)}{{\Pr}\left({Y}_{ij}^k=3\right)}\right]={\upbeta}_{02}^k+{\upbeta}_{12}^k\ {t}_{ij}+{\upbeta}_{22}^k\ {x}_i+{\upbeta}_{32}^k\ {x}_{ij}+{\upnu}_i^k+{\upmu}_i^k\ {t}_{ij}$$

where the coefficient vector (${\upbeta}_{12}^k,{\upbeta}_{22}^k,{\upbeta}_{32}^k$) indicates the effect of covariates when compare no, low and moderate use vs heavy use (i.e., 0, 1, 2 vs 3), while (${\upbeta}_{10}^k,{\upbeta}_{20}^k,{\upbeta}_{30}^k$) indicates the effect when compare no vs any use (i.e., 0 vs 1, 2, 3) for the k-th outcome, and the two sets of coefficient vectors are allowed to be different. The non-proportional odds model relaxes the homogeneous covariate effect assumption and offers more flexibility in substance use modeling. It is worth noting that Model (3) also allows “partial” proportional odds, where only a subset of the coefficients vary across the cumulative logits and others remain constant. For example, it is possible that the time trends for heavy use (vs no, low and moderate use) is different from the trends for no use (vs low, moderate and heavy use), i.e., only ${\upbeta}_{1c}^k$ vary across the cumulative logits while $\left({\upbeta}_{21}^k,{\upbeta}_{22}^k\right)$ remain the same. The “partial” proportional odds model is a special case of the non-proportional model.

## Results

Here we describe the use of multivariate mixed cumulative logit model in analyzing the substance use data. In the example data set, the usage levels of cigarettes, alcohol and marijuana were collected for 1263 subjects across 8 measurement waves (baseline, 6, 15, 24, 48, 60, 72 and 84 months). For each substance, the usage level was recoded as a 5-level ordinal outcome. Cigarette use was characterized by the number of days smoked during the last 30 days (0 = 0 days, 1 = 1–3 days, 2 = 4–10 days, 3 = 11–20 days, 4 = 21+ days). Both alcohol and marijuana use were characterized by the level of use in the past 3 months (0 = 0 times, 1 = once a month or less, 2 = > 1 a month but < 1 a week, 3 = > 1 a week but not daily, 4 = everyday). Therefore, for all three substances, the 0 category represents no use, while the highest category represents daily or near-daily use. Overall, subjects provided an average of 6.8 observations (per substance) across waves, with 87% of subjects providing 5 or more observations. Detailed breakdown of the expanded age brackets, attrition and raw breakdowns for the outcome variables are provided in Additional file 1: Table A1.1 to A1.3 in Appendix A1.

As recommended in McArdle [18] and others, we use age instead of study wave as our time variable. Observations are binned into half-year age intervals from 13.5–14.0 years at the low end, to 26.0–26.5 years at the high end (i.e., a total of 25 half-year age bins). We then treat age in years, relative to the first bin, as our age variable (0 to 13 years) in the analysis.

**Fig. 1:**
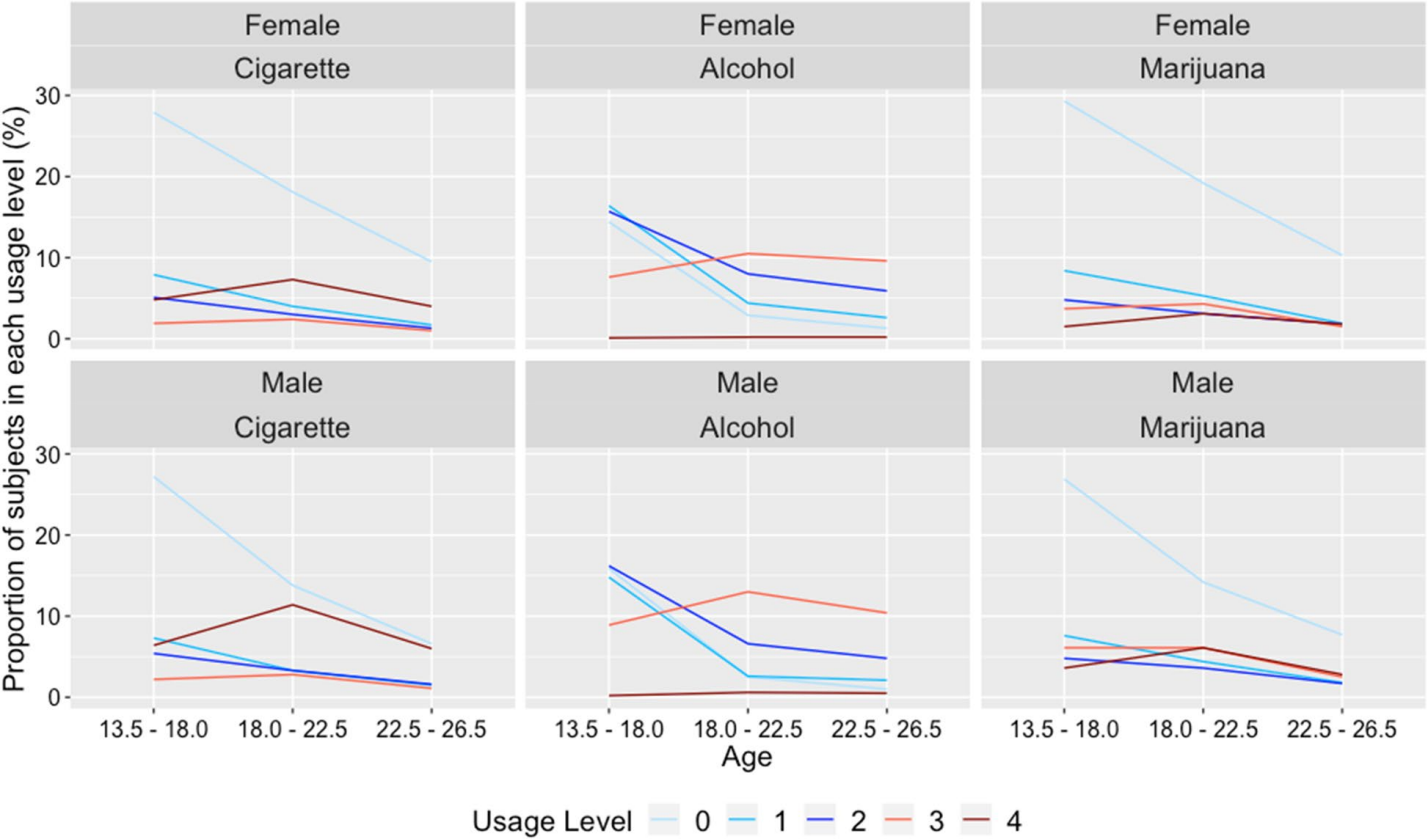
Proportion of males and females in each substance usage level and age band. Usage level 0 indicates never use, and level 4 indicates daily or near-daily use

Figure 1 shows the proportion of subjects in each age band and usage level category, tabulated for males and females, respectively. In general, as subjects grow older, the proportions of individuals in 0, 1 and 2 categories (corresponding to no, low and moderate use) decrease for both males and females, while those for categories 3 and 4 (corresponding to heavy and near-daily use) first increase and then decrease, indicating that proportional odds assumption might not hold for this data set. In addition, the decrease of individual proportion in category 1 (no use), as well as the increase in category 4 (near-daily use) seem to be sharper for males than for females, indicating possible interaction effects between gender and age. Details containing the number and proportion of males and females in each age band and usage level category are provided in Additional file 1: Table A2.1 of the Appendix A2. Statistical test results for proportional odds assumption are provided in Additional file 1: Table A3.1 of the Appendix A3.

**Fig. 2:**
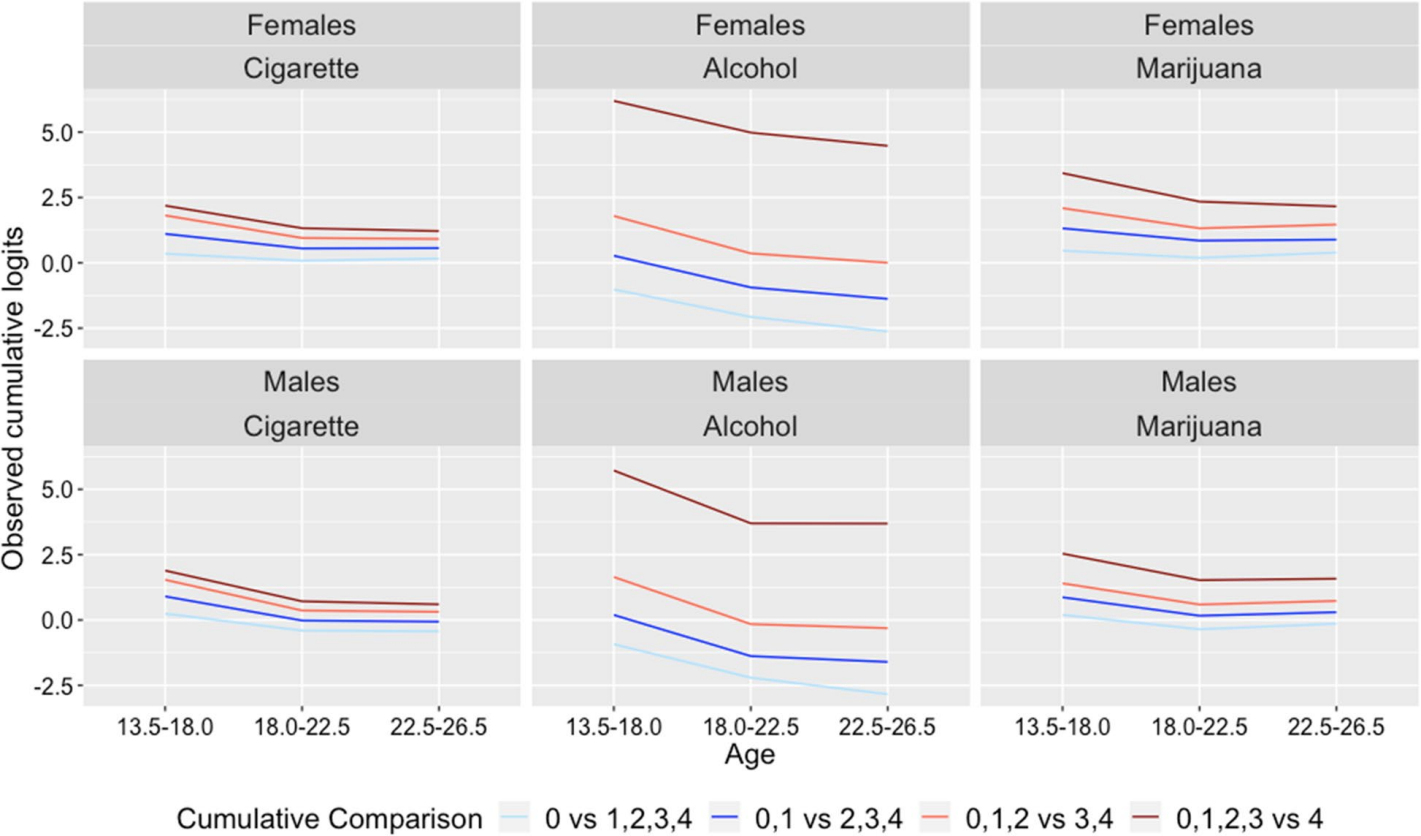
Observed cumulative logits for all cumulative comparisons and substance use over age, for males and females. Usage level 0 indicates never use, and level 4 indicates daily or near-daily use

To better illustrate the implications from Fig. 1, observed cumulative logits from “no vs any use” (0 vs 1,2,3,4) to “no to heavy use vs daily or near-daily use” (0,1,2,3 vs 4) were plotted for males and females, under each substance use and age band. The first cumulative logit compared the possibility of a 0 category vs those for 1,2,3 and 4 categories, i.e., no use vs any use, while the last cumulative logit compared the possibility of 0,1,2 and 3 categories vs that for category 4, i.e., no use to heavy use vs near-daily or daily use. As Fig. 2 indicates, there is clearly an age / time effect since all cumulative comparisons decreased with age. However, for all substance use and all cumulative comparisons, males had sharper decrease from “13.5 - 18.0” to “18.0 – 22.5”, while shallower decrease from “18.0–22.5” to “22.5 – 26.5”, compared to females, indicating differential time trends between genders and potential interaction effects between age and gender. In addition, different cumulative comparisons showed heterogeneous time trends, with less reduction over age for the first cumulative comparison (0 vs 1,2,3,4), and more reduction for the last cumulative comparison (0,1,2,3 vs 4). Details about the cumulative odds and logits for all cumulative comparisons are provided in Additional file 1: Table A4.1 of the Appendix A4.

To formally examine these implications, consider the multivariate random slope model:

4 $${\log}\left[\frac{{\Pr}\left({Y}_{ij}^k\le c\right)}{1-{\Pr}\left({Y}_{ij}^k\le c\right)}\right]={\upbeta}_{0c}^k+{\upbeta}_{1c}^k\ {age}_{ij}+{\upbeta}_{2c}^k\ {gender}_i+{\upbeta}_{3c}^k\ {age}_{ij}\ast {gender}_i+{\upnu}_i^k+{\upmu}_i^k\ {age}_{ij}$$

where c = 0,1,2,3. ${\upbeta}_{0c}^k$ indicates the fixed-effects intercept for the c-th cumulative comparison of the k-th outcome; ${\upbeta}_{1c}^k$ and ${\upbeta}_{2c}^k$ indicate the effects of age and gender; ${\upbeta}_{3c}^k$ indicates the interaction effect of age and gender, i.e., the differential time trends for males and females; ${\upnu}_i^k$ is the random subject intercept, indicating the influence of subject i on the cumulative logits at baseline, while ${\upmu}_i^k$ is the random subject slope, indicating the influence of subject i on the change of cumulative logits over time for the k-th outcome. The dependence of fixed effects parameters β on c, i.e., the subscript of c in β, relaxes the proportional odds assumption and provides separate effects estimation for each cumulative comparison. Utilizing the multivariate approach, we assume that ${W}_i=\left({\upnu}_i^1,{\upmu}_i^1,{\upnu}_i^2,{\upmu}_i^2,\dots, {\upnu}_i^K,{\upmu}_i^K\right)$ follows the 2 K dimensional multivariate Gaussian distribution as described in Model (1), allowing correlation among the usage levels in cigarettes, alcohol and marijuana.

**Fig. 3:**
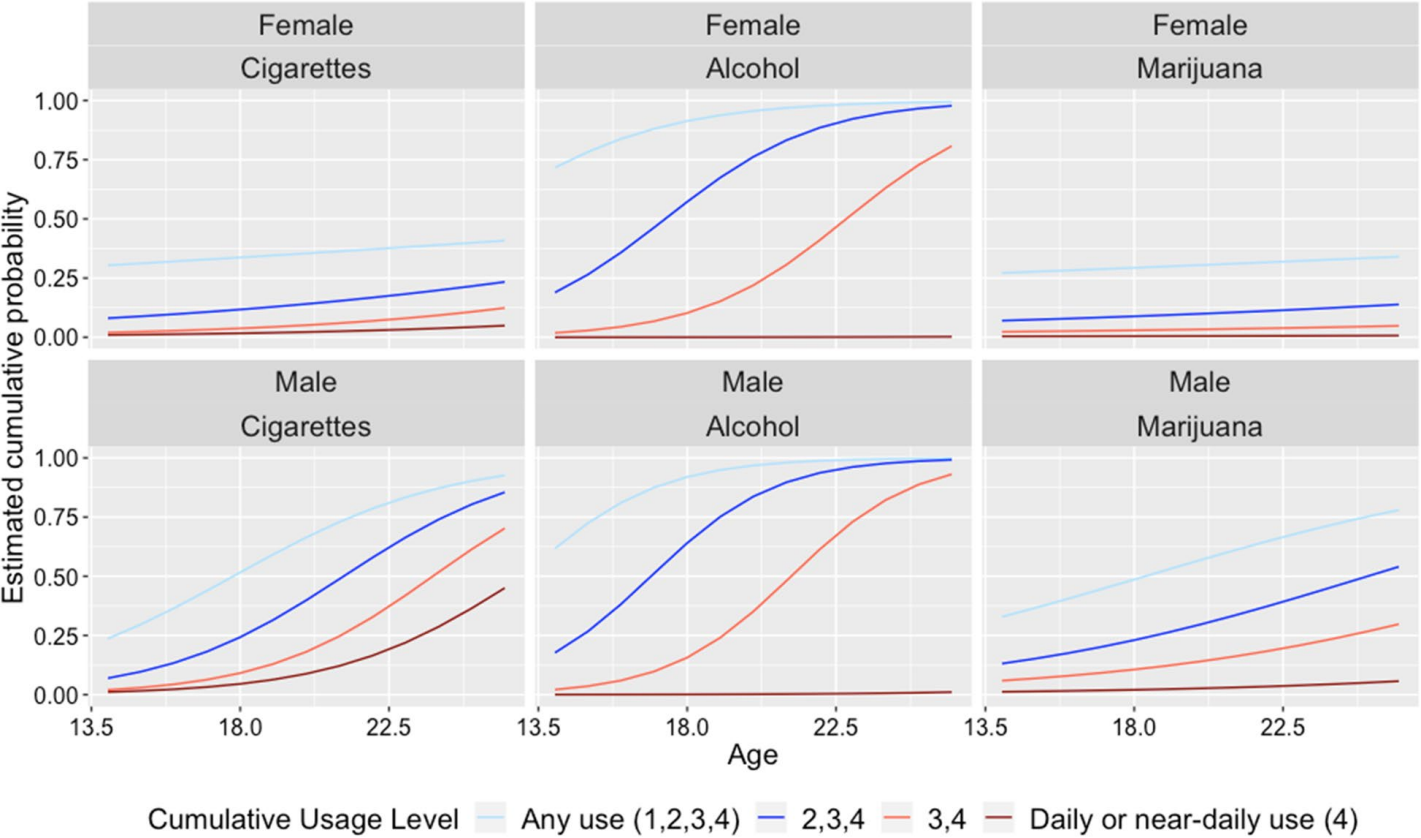
Estimated cumulative probability of substance use from any use (highest curve) to daily or near-daily use (lowest curve) for females and males over time

Parameter estimates are performed in SuperMix [19], which uses full maximum likelihood estimation. Full estimation results, including parameter estimates, standard errors and *p*-values for each cumulative logit model and each substance is provided in Additional file 1: Appendix A5. For space and visualization, we provide Fig. 3 below of the estimated cumulative probabilities for the three substances by gender. In each subplot, the highest logistic curve represents the cumulative probability of any use (categories 1 to 4 vs category 0, or equivalently, *Pr*(*Y* ≥ 0)), and the lowest logistic curve represents the cumulative probability of daily or near-daily use (categories 4 vs categories 0 to 3, or equivalently, *Pr*(*Y* ≥ 4)). The two intermediate curves can be thought to represent low and moderate use, respectively. Thus, going from top to bottom, the curves represent increasing levels of substance use.

As depicted in Fig. 3, it is clear that there are significant differences between males and females in terms of substance use across time. These gender differences are almost entirely in terms of the age trends (i.e., slopes due to age), with males having steeper trends on all curves with the exception of the daily use trends (lowest curve) for alcohol and marijuana. These gender differences in trends are more pronounced for cigarettes and marijuana, and less so for alcohol. Thus, while both gender groups have relatively similar use levels at baseline (age 13.5–14.0), large gender differences emerge as age increases. Both gender groups have increasing slopes due to age for all curves and substances, except that females show non-significant or minimally increasing trends for all levels of marijuana use, and for any use of cigarettes (highest curve). Concerning daily or near-daily use (the lowest curve), these were relatively flat with the noted exception of cigarettes for males, which showed a rather dramatic increase across age. For all others, the probability of daily use remained low. Contrasting the different substances, it is clear that the patterns for alcohol, especially, are quite different. Interestingly, alcohol showed the highest levels of any, low, or moderate use (top three curves, respectively), but the lowest levels of daily use (lowest curve), relative to cigarettes and marijuana.

In addition, Model (4) allows one to examine the inter-substance association, i.e., the associations among substance use, in terms of the random subject intercept and age effects. Table 1 shows the estimated correlation matrix for the 6 random effects: random subject intercept and slope for cigarettes (CigInt, CigAge), alcohol (AlcInt, AlcAge), and marijuana (MarijInt, MarijAge). The correlations of intercepts are all large and positive, with the strongest association between baseline alcohol and marijuana use (r = 0.804). Similarly, the age effects are positively associated, though not quite as large, with the strongest association between age-related changes in alcohol and marijuana (r = 0.503). All associations between intercepts and age effects are negative, meaning that subjects with lower/higher initial use have greater/lesser age effects. This is likely due to ceiling effects of measurement, meaning that subjects with higher initial levels cannot increase their levels to the same degree as subjects with lower initial levels.

**Table 1:** Estimated correlation of random intercept and age effects for cigarette, alcohol and marijuana use

	CigInt	CigAge	AlcInt	AlcAge	MarijInt	MarijAge
CigInt	1	–	–	–	–	–
CigAge	−0.277	1	–	–	–	–
AlcInt	0.659	−0.125	1	–	–	–
AlcAge	−0.459	0.198	−0.607	1	–	–
MarijInt	0.729	−0.155	0.804	−0.504	1	–
MarijAge	−0.326	0.400	−0.339	0.503	−0.415	1

## Discussion

In this paper, we have described a multivariate approach for analyzing longitudinal substance use data with a focus on mixed cumulative logit models with non-proportional odds assumption. Our goal is to relax the proportional odds assumption which is widely adopted by many statistical models. Proportional odds ordinal models assume homogeneous covariate effect across all cumulative comparisons, which, however, might not be appropriate in the context of substance use research. For example, a potential intervention strategy might be able to decrease substance use from the middle category, but not at the highest outcome category. In dealing with ordinal substance use data in practice, issues often arise as where to dichotomize the ordinal outcomes. For example, whether low use of cigarettes is defined as 1–3 days of smoking in the last 30 days, or 1–5 days of smoking in the last 30 days, would impact the proportional odds models since these models only estimate one set of covariate effect for all cumulative logits. The non-proportional odds cumulative logit model presented in this paper overcomes this issue by estimating one set of covariate effect for each cumulative comparison and thus solves the issue caused by inconsistent dichotomizing thresholds. Testing whether a covariate has homogeneous effect across all cumulative comparisons is sometimes of particular interest, and when proportionality cannot be assumed, our method provides a practical alternative. Brant [20] constructed a number of goodness-of-fit measures for assessing the proportional odds assumption and provided a data example for illustration.

Another advantage of our proposed model is the jointly modeling of multiple substances via random effects. The proposed multivariate approach allows both the inter-substance correlation of the usage levels and the correlation of baseline usage as well as its change over time. For inter-substance correlation, the usage level of one substance (such as cigarettes) is often associated with that of another substance (such as alcohol or marijuana) for an individual. This is likely due to person specific behavior or traits that cannot be observed from the data. Including subject random effects for each substance and allow them to be correlated provides a subject specific modeling strategy and allows the estimation of subject-specific as well as substance-specific covariate effects. The proposed model includes both subject level random intercept and slope for each substance, and allow them to be correlated both for a specific substance and across substances. Correlation between the baseline usage level and its change over time is often observed for survey data and is sometimes called the ceiling effects, which describes the phenomenon that subjects with higher/lower levels at baseline cannot increase/decrease their levels to the same degree compared to those with lower/higher initial levels. The estimated covariance matrix (or correlation matrix) for the multi-dimensional random effects provides a quantitative measurement for the inter-substance association as well as the association between baseline usage and change over time.

In the example data set, individuals were measured at up to 8 waves during the entire study. Modeling the substance use outcomes via mixed effects model framework does not require balanced data, i.e., individuals are not required to be measured at every measurement wave. Compared to fixed effects models, both information of that individual and individuals in the entire data set were used (but were weighted differently) in estimating the subject specific covariate effects. The information borrowing across all individuals makes the effect estimates more robust in the random effect approach. Using terminologies from the multi-level analysis, the multivariate longitudinal data in our example are structured with level 1 occasions and level 2 subjects, where observations (level 1) across multiple occasions are nested within subjects (level 2). The same multivariate approach is thus applicable to cross-sectional clustered data, where the level 1 observations are clustered within the level 2 clusters (such as hospitals and classrooms). However, in this situation, only random intercept model will be considered since observations are not measured repeatedly over time.

Parameter estimation in the proposed multivariate mixed cumulative logit model is challenging due to the inclusion of multiple random effects and non-proportionality. We provide a sample SuperMix code for non-proportional odds model in the Additional file 1: Appendix A6. Since multivariate longitudinal studies are increasingly used for substance use and behavioral studies, it is of great importance to develop appropriate statistical models that can help to interpret the associations and shed light on possible mechanisms.

## Conclusion

The proposed multivariate mixed cumulative logit model offers the most flexibility in jointly modeling multiple substance use longitudinally over time. Analyses of the P01 data set using the proposed model revealed differential time trend of substance use between males and females, as well as the associations among cigarettes, alcohol and marijuana use both at baseline and longitudinally over time.

## Supplementary Information

**Additional file 1.**

## Data Availability

The datasets analyzed during the current study are not publicly available due to the reason that the study is still ongoing, but are available from Dr. Robin Mermelstein on reasonable request.
