# Supplementary material for: Analysis of multivariate longitudinal substance use outcomes using multivariate mixed cumulative logit model
Source: BMC Med Res Methodol. 2021 Nov 6;21:239. doi: 10.1186/s12874-021-01444-1 (PMC8571881; doi:10.1186/s12874-021-01444-1)
Supplement: Supplementary file 1 — Additional file 1. [file 12874_2021_1444_MOESM1_ESM.docx]

**Analysis of Multivariate Longitudinal Substance Use Outcomes using Multivariate Mixed Cumulative Logit Model - Appendix**

Xiaolei Lin^1*^, Robin Mermelstein^2^, Donald Hedeker^3^

1. School of Data Science, Fudan University, China

2. Institute for Health Research and Policy, University of Illinois at Chicago, USA

3. Department of Public Health Sciences, University of Chicago, USA

*Corresponding: Xiaolei Lin, xiaoleilin@fudan.edu.cn

Appendix A1: Details about the data

Table A1.1. Number (percent) of individuals in the expanded age brackets at baseline.

| Age | 13.9-14.4 | 14.5-14.9 | 15.0-15.4 | 15.5-15.9 | 16.0-16.4 | 16.5-16.9 | 17.0-17.4 | 17.5-17.9 |
| --- | --- | --- | --- | --- | --- | --- | --- | --- |
| n (%) | 15 (1.2) | 181 (14.3) | 325 (25.7) | 315 (24.9) | 293 (23.2) | 123 (9.7) | 10 (0.8) | 1 (0.08) |

Table A1.2. Number (percent) of individuals in their corresponding drop-out wave.

| wave (month) | 0 | 6 | 15 | 24 | 48 | 60 | 72 | 84 |
| --- | --- | --- | --- | --- | --- | --- | --- | --- |
| n (%) | 30 (2.4) | 17 (1.3) | 8(0.6) | 23 (1.8) | 45 (3.6) | 24 (1.9) | 51 (4.0) | 1065 (84.3) |

Table A1.3. Number of individuals in each usage level category for cigarette, alcohol and marijuana.

| Usage level | 0 | 1 | 2 | 3 | 4 |
| --- | --- | --- | --- | --- | --- |
| Cigarette | 694 | 275 | 148 | 48 | 97 |
| Alcohol | 340 | 417 | 350 | 155 | 1 |
| Marijuana | 781 | 216 | 123 | 102 | 38 |

Appendix A2: Proportion of subjects by usage level and age category

Table A2.1. Proportion of subjects by usage level and age category, for females and males.

|  | Female | | | | | Male | | | | |
| --- | --- | --- | --- | --- | --- | --- | --- | --- | --- | --- |
| Cigarette | 0 | 1 | 2 | 3 | 4 | 0 | 1 | 2 | 3 | 4 |
| 13.5 – 18.0 | 27.9%  (1444) | 7.9%  (407) | 5.1%  (266) | 1.9%  (97) | 4.8%  (248) | 27.2%  (1022) | 7.3%  (275) | 5.4%  (204) | 2.2%  (83) | 6.4%  (239) |
| 18.0 – 22.5 | 18.1%  (938) | 4.0%  (207) | 3.0%  (157) | 2.4%  (124) | 7.3%  (379) | 13.8%  (519) | 3.3%  (123) | 3.3%  (123) | 2.8%  (106) | 11.4%  (426) |
| 22.5 – 26.5 | 9.5%  (490) | 1.7%  (87) | 1.3%  (69) | 1.0%  (52) | 4.0%  (207) | 6.6%  (249) | 1.5%  (57) | 1.6%  (59) | 1.1%  (43) | 6.0%  (224) |
|  | | | | | | | | | | |
| Alcohol | 0 | 1 | 2 | 3 | 4 | 0 | 1 | 2 | 3 | 4 |
| 13.5 – 18.0 | 14.4%  (653) | 16.4%  (744) | 15.7%  (712) | 7.6%  (346) | 0.1%  (5) | 15.9%  (520) | 14.8%  (485) | 16.2%  (532) | 8.9%  (291) | 0.2%  (6) |
| 18.0 – 22.5 | 2.9%  (132) | 4.4%  (198) | 8.0%  (363) | 10.5%  (475) | 0.2%  (8) | 2.5%  (82) | 2.6%  (84) | 6.6%  (215) | 13.0%  (426) | 0.6%  (20) |
| 22.5 – 26.5 | 1.3%  (60) | 2.6%  (119) | 5.9%  (266) | 9.6%  (434) | 0.2%  (10) | 1.0%  (34) | 2.1%  (69) | 4.8%  (157) | 10.4%  (340) | 0.5%  (15) |
|  | | | | | | | | | | |
| Marijuana | 0 | 1 | 2 | 3 | 4 | 0 | 1 | 2 | 3 | 4 |
| 13.5 – 18.0 | 29.3%  (1509) | 8.4%  (430) | 4.8%  (248) | 3.7%  (193) | 1.5%  (77) | 26.9%  (1006) | 7.6%  (285) | 4.8%  (179) | 6.1%  (228) | 3.6%  (134) |
| 18.0 – 22.5 | 19.2%  (987) | 5.3%  (275) | 3.1%  (160) | 4.3%  (222) | 3.1%  (158) | 14.2%  (532) | 4.4%  (166) | 3.6%  (133) | 6.1%  (229) | 6.1%  (231) |
| 22.5 – 26.5 | 10.3%  (531) | 1.9%  (99) | 1.8%  (92) | 1.5%  (75) | 1.8%  (92) | 7.7%  (286) | 1.8%  (67) | 1.7%  (62) | 2.5%  (95) | 2.8%  (105) |

Appendix A3: Brant test for proportional odds assumption for cigarette, alcohol and marijuana usage levels.

Table A3.1. Brant test for proportional odds assumption for cigarette, alcohol and marijuana usage levels.

| Substance: cigarette | | | |
| --- | --- | --- | --- |
| Test for: | chi-square | df | probability |
| Omnibus | 207.91 | 9 | 0 |
| age | 42.77 | 3 | 0 |
| gender | 9.37 | 3 | 0.02 |
| age * gender | 4.76 | 3 | 0.19 |
| Substance: alcohol | | | |
| Test for: | chi-square | df | probability |
| Omnibus | 27.2 | 9 | 0 |
| age | 3.71 | 3 | 0.29 |
| gender | 7.53 | 3 | 0.06 |
| age * gender | 0.8 | 3 | 0.85 |
| Substance: marijuana | | | |
| Test for: | chi-square | df | probability |
| Omnibus | 132.96 | 9 | 0 |
| age | 33.74 | 3 | 0 |
| gender | 26.95 | 3 | 0 |
| age * gender | 8.9 | 3 | 0.03 |

Appendix A4: Observed cumulative odds and logits by usage level and age category

Table A4.1. Observed cumulative odds (logits) by usage level and age category, for females and males.

|  | Female | | | | Male | | | |
| --- | --- | --- | --- | --- | --- | --- | --- | --- |
| Cigarette | 0 vs 1,2,3,4 | 0,1 vs 2,3,4 | 0,1,2 vs 3,4 | 0,1,2,3 vs 4 | 0 vs 1,2,3,4 | 0,1 vs 2,3,4 | 0,1,2 vs 3,4 | 0,1,2,3 vs 4 |
| 13.5 – 18.0 | 1.42  (0.35) | 3.03  (1.11) | 6.14  (1.81) | 8.93  (2.19) | 1.28  (0.24) | 2.47  (0.90) | 4.66  (1.54) | 6.63  (1.89) |
| 18.0 – 22.5 | 1.08  (0.08) | 1.73  (0.55) | 2.59  (0.95) | 3.76  (1.33) | 0.67  (-0.40) | 0.98  (-0.02) | 1.44  (0.36) | 2.04  (0.72) |
| 22.5 – 26.5 | 1.18  (0.17) | 1.76  (0.56) | 2.49  (0.91) | 3.37  (1.22) | 0.65  (-0.43) | 0.94  (-0.06) | 1.37  (0.31) | 1.82  (0.60) |
|  | | | | | | | | |
| Alcohol | 0 vs 1,2,3,4 | 0,1 vs 2,3,4 | 0,1,2 vs 3,4 | 0,1,2,3 vs 4 | 0 vs 1,2,3,4 | 0,1 vs 2,3,4 | 0,1,2 vs 3,4 | 0,1,2,3 vs 4 |
| 13.5 – 18.0 | 0.36  (-1.02) | 1.31  (0.27) | 6.01  (1.79) | 491.0  (6.20) | 0.40  (-0.93) | 1.21  (0.19) | 5.18  (1.64) | 304.67  (5.72) |
| 18.0 – 22.5 | 0.13  (-2.07) | 0.39  (-0.94) | 1.43  (0.36) | 146.0  (4.98) | 0.11  (-2.21) | 0.25  (-1.38) | 0.85  (-0.16) | 40.35  (3.70) |
| 22.5 – 26.5 | 0.07  (-2.63) | 0.25  (-1.38) | 1.00  (0.00) | 87.9  (4.48) | 0.06  (-2.84) | 0.20  (-1.6) | 0.73  (-0.31) | 40.0  (3.69) |
|  | | | | | | | | |
| Marijuana | 0 vs 1,2,3,4 | 0,1 vs 2,3,4 | 0,1,2 vs 3,4 | 0,1,2,3 vs 4 | 0 vs 1,2,3,4 | 0,1 vs 2,3,4 | 0,1,2 vs 3,4 | 0,1,2,3 vs 4 |
| 13.5 – 18.0 | 1.59  (0.46) | 3.74  (1.32) | 8.10  (2.09) | 30.91  (3.43) | 1.22  (0.20) | 2.39  (0.87) | 4.06  (1.40) | 12.67  (2.54) |
| 18.0 – 22.5 | 1.21  (0.19) | 2.34  (0.85) | 3.74  (1.32) | 10.41  (2.34) | 0.70  (-0.36) | 1.18  (0.16) | 1.81  (0.59) | 4.59  (1.52) |
| 22.5 – 26.5 | 1.48  (0.39) | 2.43  (0.89) | 4.32  (1.46) | 8.66  (2.16) | 0.87  (-0.14) | 1.35  (0.30) | 2.08  (0.73) | 4.86  (1.58) |

Appendix A5: Coefficient Estimates

Cigarette use (estimate, std err, p-value)

logit Females Males Difference

1 intercept -0.809 0.329 0.014 -1.016 0.272 0.001

1 age 0.038 0.029 0.190 0.309 0.034 0.001 ***

2 intercept -2.383 0.320 0.001 -2.412 0.258 0.001

2 age 0.104 0.030 0.001 0.364 0.035 0.001 ***

3 intercept -3.808 0.360 0.001 -3.683 0.304 0.001

3 age 0.161 0.033 0.001 0.395 0.036 0.001 ***

4 intercept -4.550 0.996 0.001 -4.293 0.975 0.001

4 age 0.138 0.036 0.001 0.356 0.038 0.001 ***

Alcohol Use

logit Females Males Difference

1 intercept 1.106 0.317 0.001 0.724 0.147 0.001 *

1 age 0.358 0.026 0.001 0.489 0.033 0.001 ***

2 intercept -1.238 0.300 0.001 -1.273 0.139 0.001

2 age 0.437 0.021 0.001 0.529 0.027 0.001 **

3 intercept -3.749 0.333 0.001 -3.561 0.157 0.001

3 age 0.451 0.022 0.001 0.536 0.025 0.001 **

4 intercept -8.896 1.124 0.001 -7.868 0.488 0.001

4 age 0.242 0.086 0.005 0.291 0.072 0.001

Marijuana Use

logit Females Males Difference

1 intercept -0.973 0.321 0.003 -0.632 0.255 0.013

1 age 0.027 0.026 0.293 0.165 0.029 0.000 ***

2 intercept -2.552 0.313 0.001 -1.805 0.239 0.001 ***

2 age 0.063 0.028 0.024 0.171 0.030 0.001 **

3 intercept -3.720 0.354 0.001 -2.687 0.284 0.001 ***

3 age 0.064 0.031 0.040 0.159 0.031 0.001 *

4 intercept -5.585 1.002 0.001 -4.335 0.972 0.001 ***

4 age 0.057 0.041 0.163 0.133 0.037 0.001

logit 1: any use * p < .05

logit 2: low use and above ** p < .01

logit 3: moderate use and above *** p < .001

logit 4: daily use

Appendix A6: SuperMix code

[Main]

Type=Mixed Up Model Settings

DataFile=U:\workdir\CigPotAlcAgeSexdat.ss3

Version=2.1

ModelType=ordered

Region=American

[Configuration]

Title1=P01 data - Cigs, Pot, Alc across Age bins by Gender

Title2=Random Int & Age on all

Level2ID=1

Level3ID=-1

DepVarType=1

DepVar=2

DepVarCatNum=5

DepVarCats=1, 2, 3, 4

WriteBayes=0

ConvCritera=0.0001

EMiterations=100

RepVarTerms=1

AutoCor=0

ErrorForm=0

AutoTerms=0

OutputType=0

UnitWeight=0

WeightCol1=-1

WeightCol2=-1

WeightCol3=-1

FuncModel=1

OptMeth=1

NumQuad=5

Quadrature=0

Prior=0

[Variables]

FixedNum=11

FixedNumZI=0

FixedInt=1

FixedIntZI=0

Fixed=5, 7, 6, 8, 10, 12, 13, 14, 15, 16, 17

FixedZI=

Random2Num=6

Random2NumZI=0

Rand2Int=0

Rand2IntZI=0

Random2=5, 6, 7, 8, 9, 10

Random2ZI=

Random3Num=1

Random3NumZI=0

Rand3Int=1

Rand3IntZI=0

Random3=

Random3ZI=

GenerateMeans=0

MeansCol=-1

TimeCol=-1

GenerateCrossTab=0

CrossTabCol=-1

[StartingValues]

Starting=0

ErrorVariance=1

StartFixed=0, 0, 0, 0, 0, 0, 0, 0, 0, 0, 0

StartCov2=1, 0, 1, 0, 0, 1, 0, 0, 0, 1, 0, 0, 0, 0, 1, 0, 0, 0, 0, 0, 1

StartCov3=,

Thresholds=3

Thresh1=, , , , , , , , , , ,

Thresh2=, , , , , , , , , , ,

Thresh3=, , , , , , , , , , ,

StartAuto=, , , , , , , , , , , , , , , , , , , , , , , , , , , , , , , , , , , , , , , , , , , , , , , , , , , , , , , , , , , , , , , , , , , , , , , , , , , , , , , , , , , , , , , , , , , , , , , , , , , , , , , , , , , , , , , , , , , , , , , , , , , , , , , , , , , , , , , , , , , , , , , , , , , , , , , , , , , , , , , , , , , , , , , , , , , , , , , , , , , , , , , , , , , , , , , , , , , , , , , , , , , , , , , , , , , , , , , , , , , , , , , , , , , , , , , , , , , , , , , , , , , , , , , , , , , , , , , , , , , , , , , , , , , , , , , , , , , , , , , , , , , , , , , , , , , , , , , , , , , , , , , , , , , , , , , , , , , , , , , , , , , , , , , , , , , , , , , , , , , , , , , , , , , , , , , , , , , , , , , , , , , , , , , , , , , , , , , , , , , , , , , , , , , , , , , , , , , , , , , , , , , , , , , , , , , , , , , , , , , , , , , , , , , , , , , , , , , , , , , , , , , , , , , , , , , , , , , , , , , , , , , , , , , , , , , , , , , , , , , , , , , , , , , , , , , , , , , , , , ,

StartThresh=, , ,

[MissingValues]

Missing=0

[Patterns]

ExpVar=0

Random2=0

Random3=0

FixPat=, , , , , , , , , , ,

Cov2Pat=, , , , , , , , , , , , , , , , , , , ,

Cov3Pat=,

[Advanced]

ExpVarIncInt=1

ExpVarInter=11

RightCensor=0

CensorCol=-1

ModelTerms=0

TimeOffset=0

OffsetCol=-1

DistModelCont=0

EstScaleCont=0

RandomThresh2=0

RandomThresh3=0

RefCat=0

DistModelCount=0

EstScaleCount=0

Dispersion=1

DistModelBinary=0

EstScaleBinary=0

NumTrials=-1

[Transforms]

Number=0

NumExpVar=1

ExpVar=1, 1, 1, 3,

NumRandom2=1

Random2=1, 1, 1, 3,

NumRandom3=1

Random3=1, 1, 1, 3,

NumThresholds=1

Thresholds=1, 1, 1, 3,

NumInteractions=1

Interactions=1, 1, 1, 3,
